# Supplementary material for: Direct interaction between TDP-43 and Tau promotes their co-condensation, while suppressing Tau fibril formation and seeding
Source: EMBO J. 2025 Oct 17;44(24):7395–433. doi: 10.1038/s44318-025-00590-2 (PMC12706024; doi:10.1038/s44318-025-00590-2)
Supplement: Supplementary file 6 — Expanded View Figures [file 44318_2025_590_MOESM6_ESM.pdf]

Expanded View Figures

Figure EV1. pTau, but not the Tau N-terminal domain or repeat domain, enriches in TDP-43 condensates and aggregates.

(**A**) Scheme of recombinant Tau variants with their respective net charges; created with BioRender.com. (**B**) Coomassie-stained SDS-PAGE gel showing the molecular sizes of Tau, phosphorylated Tau (pTau), Tau N-terminal domain (Tau-NTD), and the Tau-RD fragment (R1–R4 repeat domains). (**C**) Confocal microscopy images of Alexa488-labeled TDP-43 (5 µM) in absence or presence of DyLight650-labeled Tau, pTau, Tau-NTD, or Tau-RD in a phase separation assay. Scale bar: 15 µm in overview and 3 µm in inset. (**D**) Colocalization of Tau, pTau, Tau-NTD, or Tau-RD within TDP-43 condensates quantified by measuring the area of condensates exhibiting overlapping green and far-red signals. Quantification was performed across ($n = 3$) biological replicates, and bar graphs show values ± SEM. (**E**) Confocal microscopy images of Alexa488-labeled TDP-43 (5 µM) in absence or presence of DyLight650-labeled Tau, pTau, Tau-NTD, or Tau-RD in an aggregation assay. Scale bar: 15 µm in overview and 3 µm in inset. (**F**) Colocalization of Tau, pTau, Tau-NTD, or Tau-RD within TDP-43 aggregates quantified by measuring the area of aggregates exhibiting overlapping green and far-red signals. Quantification was performed across ($n = 3$) biological replicates, and bar graphs show values ± SEM.

© The Author(s)

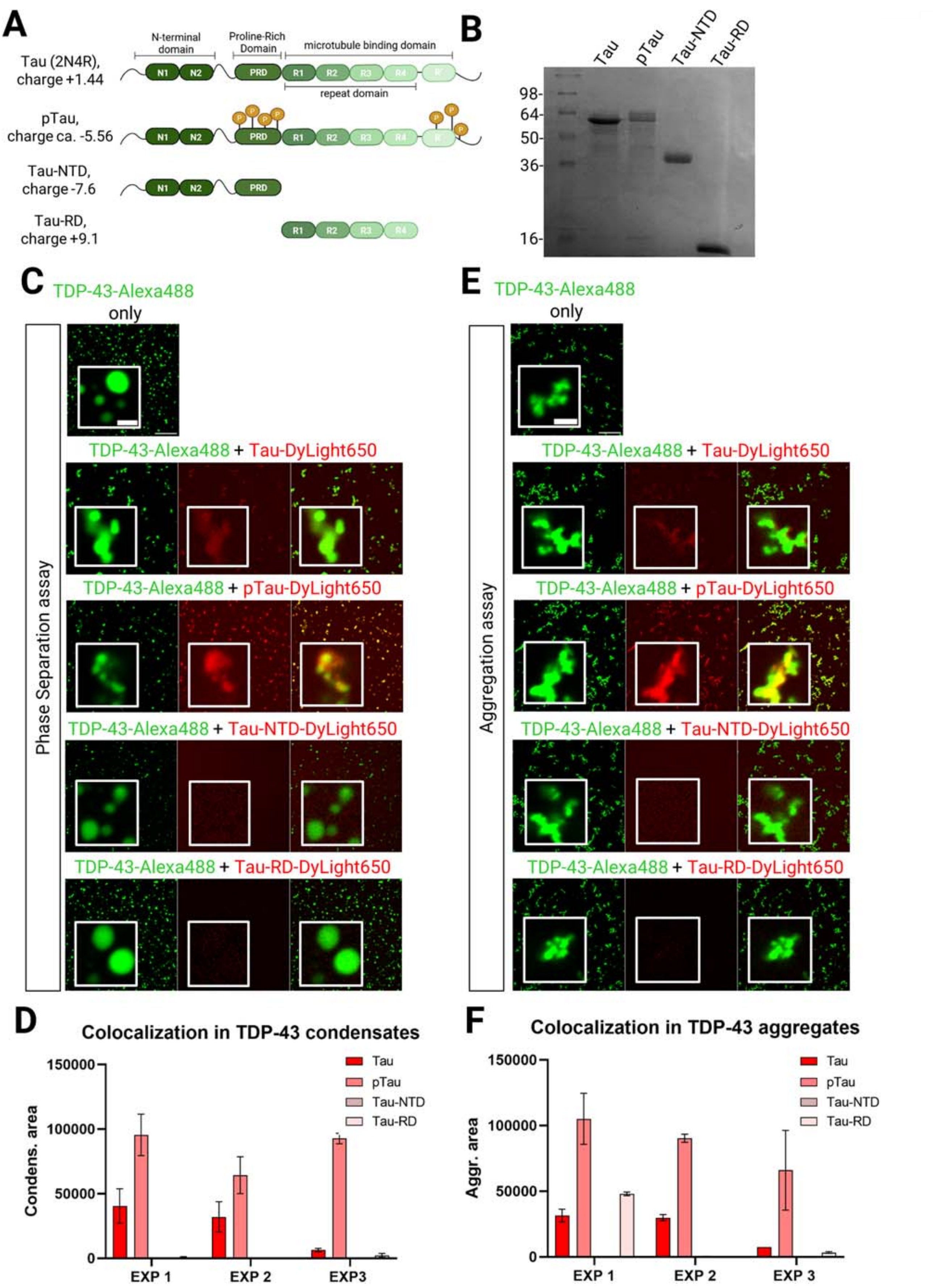
A
N-terminal domain
Proline-Rich Domain
microtubule binding domain
repeat domain
Tau (2N4R), charge +1.44
pTau, charge ca. -5.56
Tau-NTD, charge -7.6
Tau-RD, charge +9.1
B
Tau
pTau
Tau-NTD
Tau-RD
98-
64-
50-
36-
16-
C
TDP-43-Alexa488 only
Phase Separation assay
TDP-43-Alexa488 + Tau-DyLight650
TDP-43-Alexa488 + pTau-DyLight650
TDP-43-Alexa488 + Tau-NTD-DyLight650
TDP-43-Alexa488 + Tau-RD-DyLight650
E
TDP-43-Alexa488 only
Aggregation assay
D
Colocalization in TDP-43 condensates
Condens. area
150000
100000
50000
0
Tau
pTau
Tau-NTD
Tau-RD
EXP 1
EXP 2
EXP3
F
Colocalization in TDP-43 aggregates
Aggr. area
EXP 1
EXP 2
EXP 3

© The Author(s)

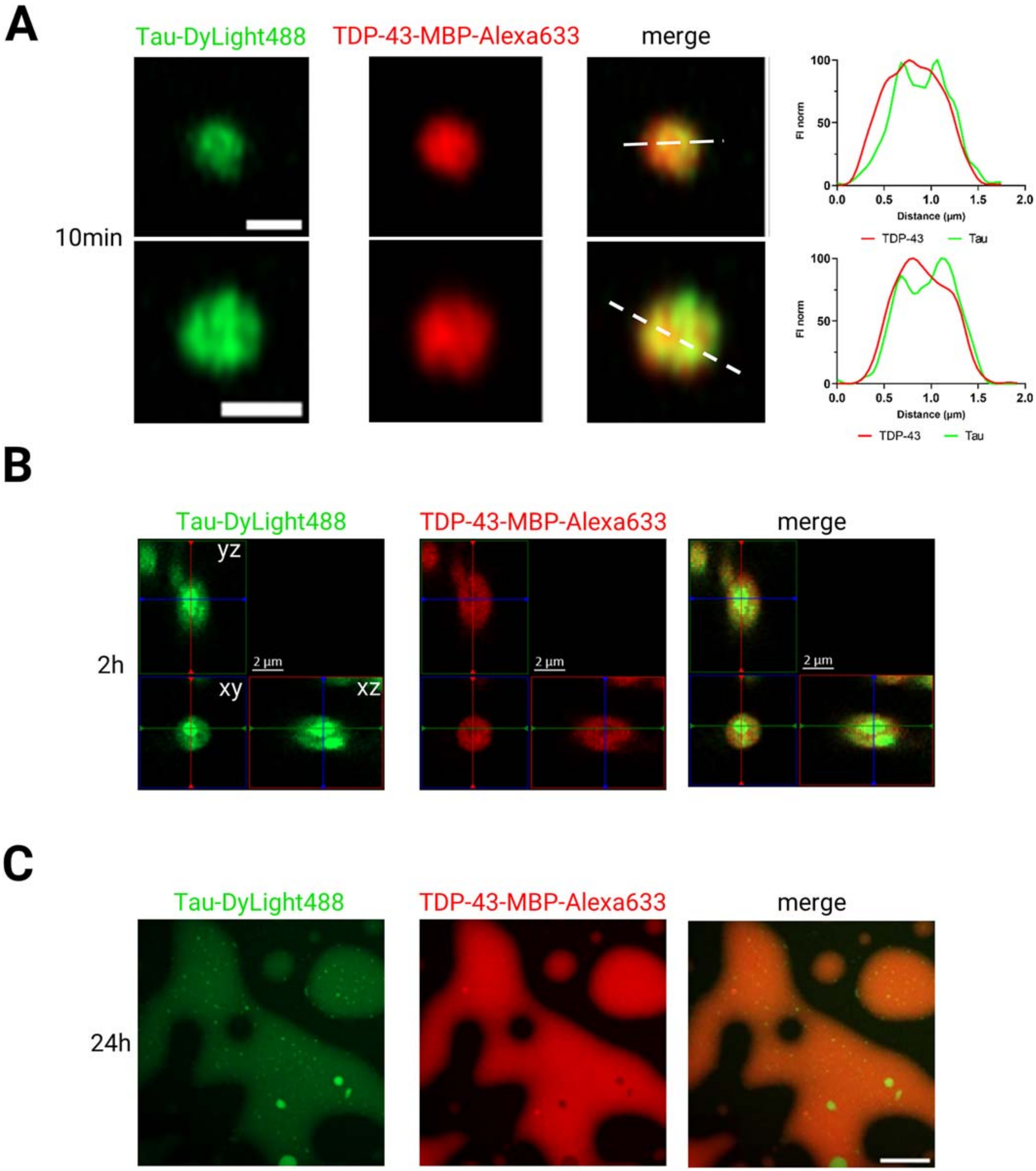

Figure EV2. Early intra-droplet structures appear at the initial stages of multiphasic Tau/TDP-43 co-condensate formation.

(**A–C**) High resolution images using AiryScan of 1:1 DyLight488-labeled Tau with Alexa633-labeled TDP-43-MBP mixed condensates after 10 min (**A**), 2 h (**B**) or (**C**) 24 h incubation. Scale bar: 1 µm (**A**), 2 µm (**B**) or 10 µm (**C**). In (**A**), dotted lines show the origin of line profiles indicated on the right, demonstrating demixing of Tau and TDP-43 within the same condensate at early timepoints.

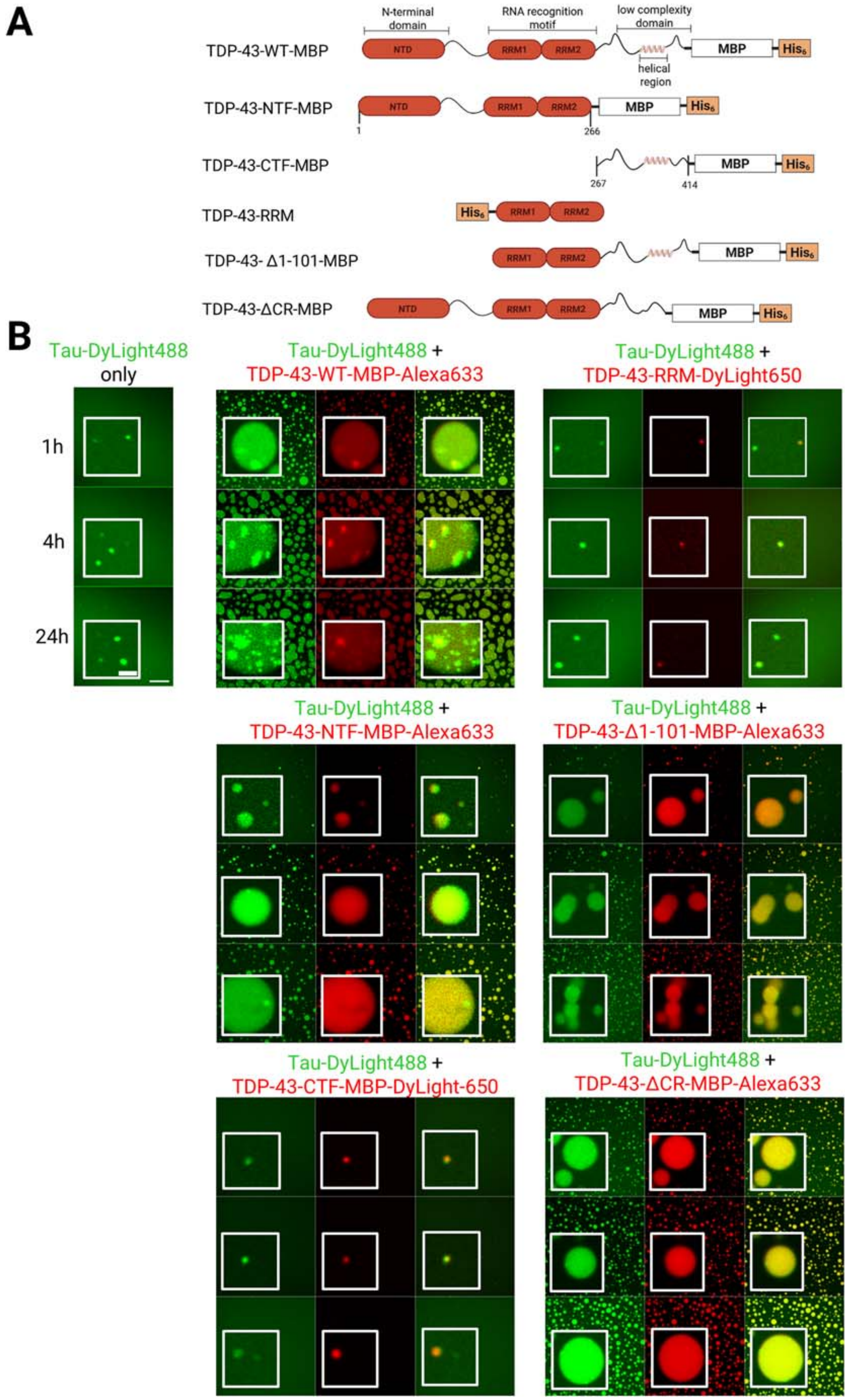
A
N-terminal domain
RNA recognition motif
low complexity domain
helical region
TDP-43-WT-MBP
TDP-43-NTF-MBP
TDP-43-CTF-MBP
TDP-43-RRM
TDP-43- Δ1-101-MBP
TDP-43-ΔCR-MBP
NTD
RRM1
RRM2
MBP
His6
1
266
267
414
B
Tau-DyLight488 only
Tau-DyLight488 + TDP-43-WT-MBP-Alexa633
Tau-DyLight488 + TDP-43-RRM-DyLight650
1h
4h
24h
Tau-DyLight488 + TDP-43-NTF-MBP-Alexa633
Tau-DyLight488 + TDP-43-Δ1-101-MBP-Alexa633
Tau-DyLight488 + TDP-43-CTF-MBP-DyLight-650
Tau-DyLight488 + TDP-43-ΔCR-MBP-Alexa633

 © The Author(s)

Figure EV3. Distinct regions of TDP-43 contribute to Tau phase separation and the formation of multiphasic co-condensates.

(**A**) Scheme of recombinant TDP-43 deletion mutants; created with BioRender.com. (**B**) Confocal microscopy images of 4 µM DyLight488-labeled Tau in equimolar presence of Alexa633-labeled TDP-43-WT-MBP, TDP-43-NTF-MBP, TDP-43-CTF-MBP, TDP-43-RRM, TDP-43-Δ1-101-MBP, or TDP-43-ΔCR-MBP at the indicated timepoints (1, 4, 24 h). Scale bar: 20 µm in overview and 2 µm in inset.

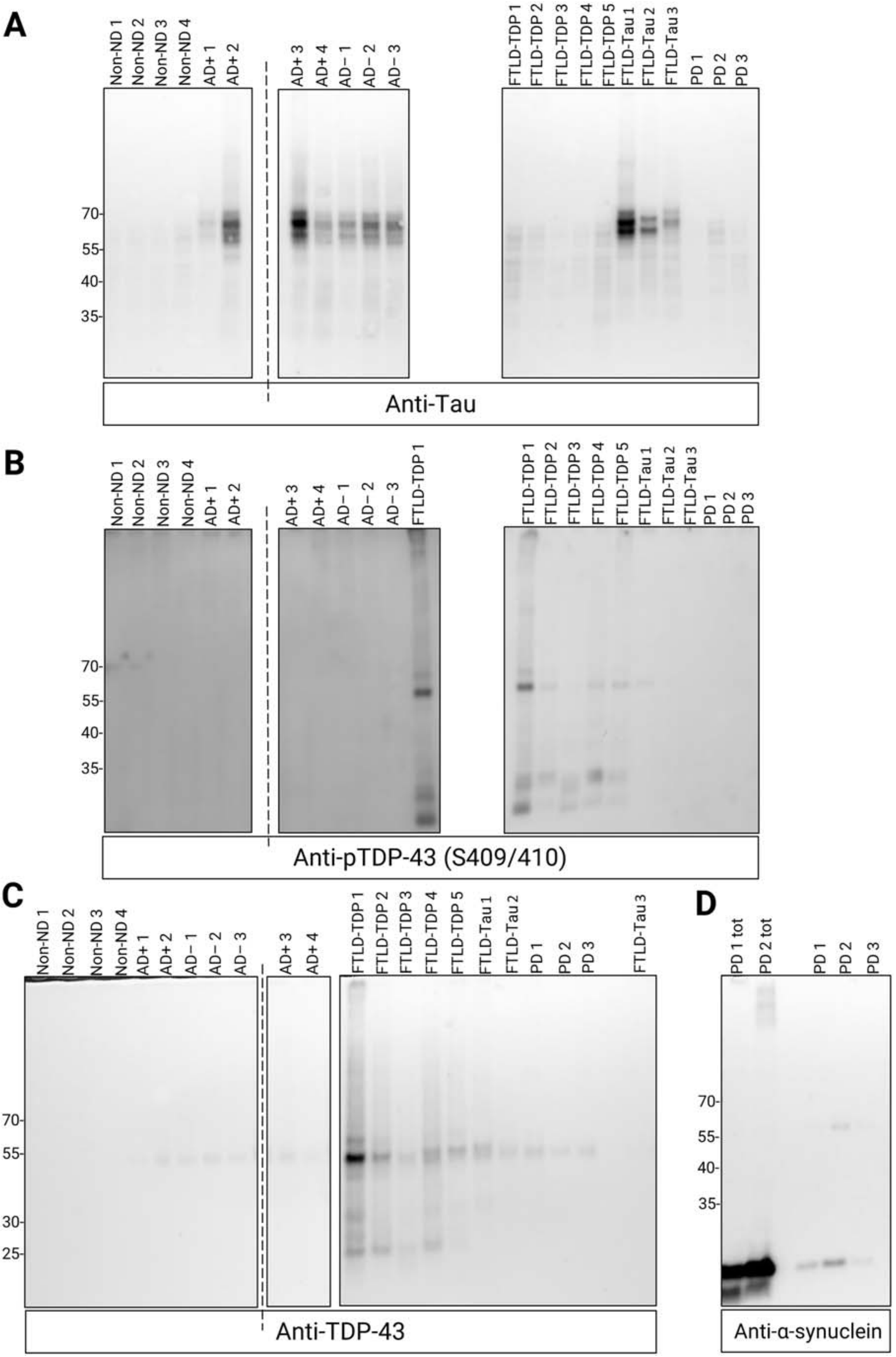
A
Non-ND 1
Non-ND 2
Non-ND 3
Non-ND 4
AD+ 1
AD+ 2
AD+ 3
AD+ 4
AD− 1
AD− 2
AD− 3
FTLD-TDP 1
FTLD-TDP 2
FTLD-TDP 3
FTLD-TDP 4
FTLD-TDP 5
FTLD-Tau 1
FTLD-Tau 2
FTLD-Tau 3
PD 1
PD 2
PD 3
70
55
40
35
Anti-Tau
B
Anti-pTDP-43 (S409/410)
C
30
25
Anti-TDP-43
D
PD 1 tot
PD 2 tot
Anti-α-synuclein

 © The Author(s)

Figure EV4. Western blot characterization of SarkoSpin extracts from human patients.

(**A**) anti-Tau antibody; (**B**) Anti-phospho-TDP-43 (S409/410) antibody; (**C**) Anti-TDP-43 antibody; (**D**) Anti-α-synuclein antibody. Western blots of SarkoSpin fractions derived from the frontal cortex of non-ND, AD +, AD–, FTLD-TDP, and FTLD-Tau patients, and from the cingulate cortex of PD patients, probed with the indicated antibodies. Dashed lines indicate divisions within the same blot.

© The Author(s)

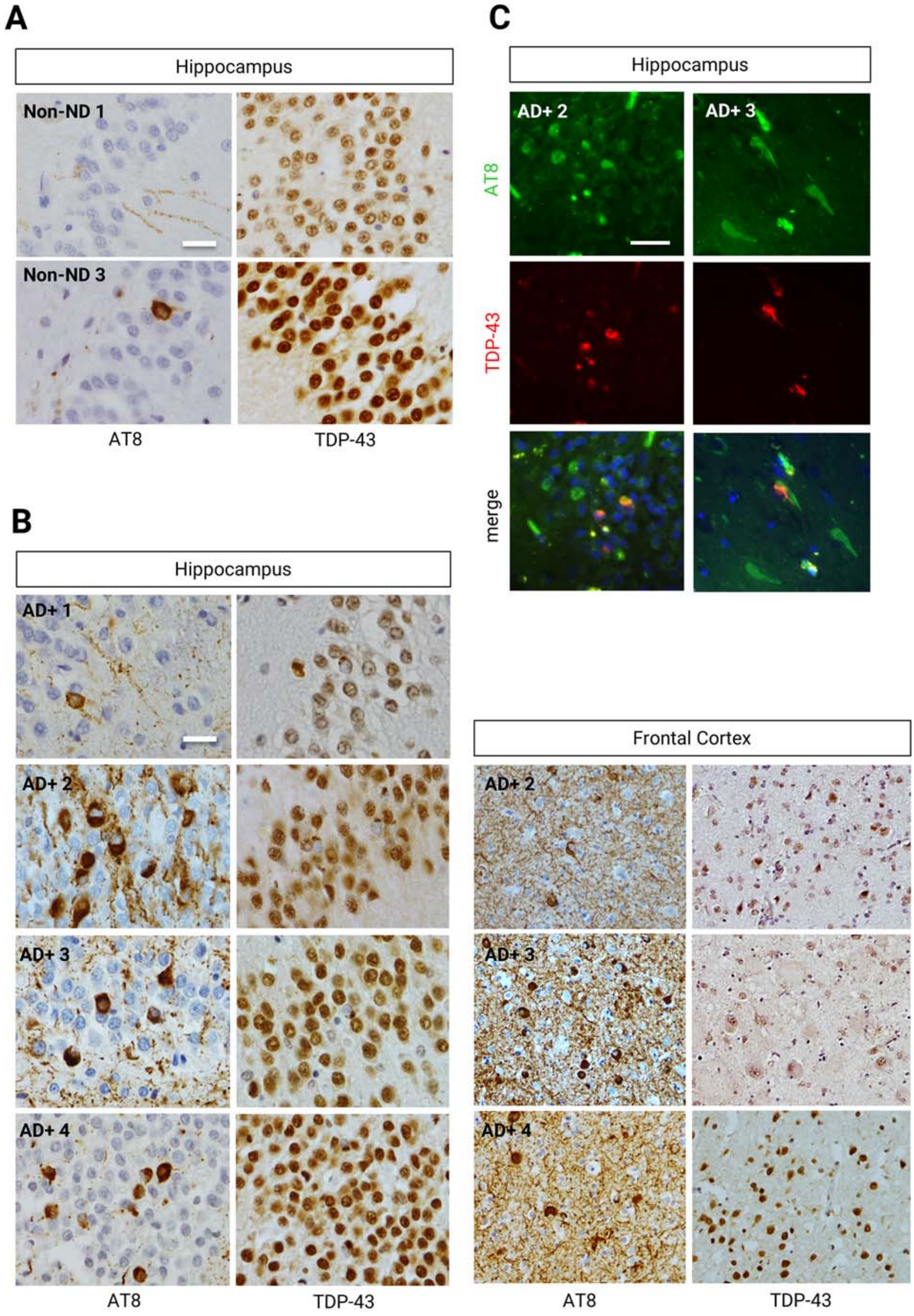
A
Hippocampus
Non-ND 1
Non-ND 3
AT8
TDP-43
B
Hippocampus
AD+ 1
AD+ 2
AD+ 3
AD+ 4
AT8
TDP-43
C
Hippocampus
AD+ 2
AD+ 3
AT8
TDP-43
merge
Frontal Cortex
AD+ 2
AD+ 3
AD+ 4
AT8
TDP-43

 © The Author(s)

Figure EV5. Characterization of patient-derived SarkoSpin extracts by immunohistochemistry and dual immunofluorescence.

(**A**, **B**) Immunohistochemical images of representative non-ND and AD+ brain sections stained with antibodies against phosphorylated Tau (AT8) and TDP-43 in either the hippocampus or frontal cortex. Scale bar: 50 µm. (**C**) Double fluorescence immunostaining of two representative AD+ cases showing phosphorylated Tau (AT8, green) and TDP-43 (red) in the hippocampus. Scale bar: 30 µm.

© The Author(s)
